# Analyses and Comparison of Accuracy of Different Genotype Imputation Methods

**DOI:** 10.1371/journal.pone.0003551

**Published:** 2008-10-29

**Authors:** Yu-Fang Pei, Jian Li, Lei Zhang, Christopher J. Papasian, Hong-Wen Deng

**Affiliations:** 1 Institute of Molecular Genetics, School of Life Science and Technology, Xi'an Jiaotong University, Xi'an, Shaanxi, People's Republic of China; 2 School of Medicine, University of Missouri-Kansas City, Kansas City, Missouri, United States of America; 3 Laboratory of Molecular and Statistical Genetics, College of Life Sciences, Hunan Normal University, Changsha, Hunan, People's Republic of China; Vrije Universiteit Medical Centre, Netherlands

## Abstract

The power of genetic association analyses is often compromised by missing genotypic data which contributes to lack of significant findings, e.g., in *in silico* replication studies. One solution is to impute untyped SNPs from typed flanking markers, based on known linkage disequilibrium (LD) relationships. Several imputation methods are available and their usefulness in association studies has been demonstrated, but factors affecting their relative performance in accuracy have not been systematically investigated. Therefore, we investigated and compared the performance of five popular genotype imputation methods, MACH, IMPUTE, fastPHASE, PLINK and Beagle, to assess and compare the effects of factors that affect imputation accuracy rates (ARs). Our results showed that a stronger LD and a lower MAF for an untyped marker produced better ARs for all the five methods. We also observed that a greater number of haplotypes in the reference sample resulted in higher ARs for MACH, IMPUTE, PLINK and Beagle, but had little influence on the ARs for fastPHASE. In general, MACH and IMPUTE produced similar results and these two methods consistently outperformed fastPHASE, PLINK and Beagle. Our study is helpful in guiding application of imputation methods in association analyses when genotype data are missing.

## Introduction

Technological advances in genotyping have increased the ability to detect dense single nucleotide polymorphisms (SNPs) in the human genome. To date, over three million SNPs have been documented by the HapMap Project [1], [2]. The availability of high-throughput genotyping has benefited biological researchers in several ways, including, improved power for genetic association analyses [3], [4]. However, challenges exist currently. For example, although the popular Affymetrix 500K Array Set contains approximately 500,000 SNPs, this only represents one sixth of the approximately three million SNPs detected by the HapMap project. Furthermore, many of these 500,000 SNPs may not be available for use in association analyses due to low call rates, deviations from Hardy-Weinberg equilibrium, rare alleles, and etc. As a result, genotype data is often missing, and this missing data results in a power loss in association studies [5]. Additionally, different platforms usually contain distinct sets of SNPs, making it difficult to replicate significant findings or to perform follow-up meta-analysis [6].

Imputation methods, used to infer missing or untyped SNP genotypes based on known information (e.g. linkage disequilibrium between missing or untyped SNPs and their flanking typed SNPs) can provide partial solutions for recovering missing or untyped genotype data [7], [8], [9], [10], [11]. Several imputation methods using various statistical models such as the haplotype-clustering algorithm [12], the hidden Markov model (HMM) [5], and the Markov Chain model [13], have been proposed. Imputed genotypes, generated with these methods, have been used, successfully, to improve power in association analyses [5], [14], [15], [16], [17], [18], [19], to facilitate meta-analyses, and to replicate significant findings in follow-up studies [6].

As new methods for genotype imputation are developed, the relative performance of these methods must be assessed. Yu and Schaid [20] compared the performance of eight genotype imputation methods [12], [21], [22], [23], [24], [25], [26] under different LD levels using real data from the HapMap project. In their study, 5% of observed genotypes were randomly set to be missing, various imputation methods were used to impute the “missing” genotypes, and the imputation error rates for the various methods were then compared. The authors concluded that fastPHASE generally had the highest accuracy among the methods tested. However, as the methodology development and use of genotype imputation continues to evolve, it has become apparent that the study of Yu et al. has significant limitations which may affect the general applicability of their conclusions, for the following reasons. First, the authors assumed that all markers were typed and that only a small fraction, (e.g., 5%), among them was missing due to genotyping failure. As imputation has evolved to the current level of resolution in which inferences can be made about genotypes at totally untyped markers, missing genotypes will account for a much larger proportion than utilized in their study; under these circumstances, their conclusions may not be applicable. Second, several additional highly effective statistical methods have been proposed for genotype imputation since the study of Yu et al., and knowledge on the relative performance of these methods, and the impact on the conclusions of the study of Yu at al., are largely unknown. Third, the authors only considered the effect of LD on imputation efficiency. Though the success of imputation is largely determined by the patterns of LD, some additional properties of the sample, such as marker density and minor allele frequency (MAF), may also influence the imputation process and performance and thus need to be investigated as we did in our study. Finally, in circumstances where markers are totally untyped, external information, (e.g., reference samples from the HapMap project), is required. Reference samples may play a central role in the success of imputation and, consequently, it is necessary to assess its effect on the imputation process, as we did in the current study.

Several additional comparisons of imputation methods have been conducted in the context of new methods being described and compared to alternative methods [7], [10], [12]. In general, however, these comparisons are fairly limited in scope and not comprehensive. Consequently, we perceived a substantial need to perform a comprehensive comparison of recently developed, sophisticated methods for imputation.

In this study, to evaluate the factors potentially affecting imputation accuracy rates (ARs), we used both simulated and real data sets to investigate the effects of LD, MAF of untyped loci, marker density, and reference sample size on the performance of five popular imputation methods: MACH, IMPUTE, fastPHASE, PLINK and Beagle. We also compared their relative performance under various conditions.

## Results

### Analyses of Simulated Data

**Figure 1:**
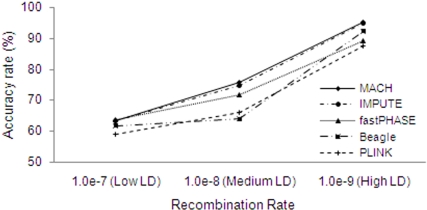
Effects of LD level on accuracy rates. The results are based on 90 reference haplotypes and a medium marker density (one SNP per 6 kb).

Figure 1, which illustrates the effects of LD level on the performance of various methods, shows that ARs increased remarkably as LD levels became stronger. For example, when the reference sample size was 90, the AR for MACH was 62.8% at the low LD level, increased to 75.9% at the medium LD level, and reached 95.1% at the high LD level. When comparing the different methods, MACH and IMPUTE performed similarly, and both produced higher ARs than alternative methods under all LD levels simulated, with the exception of fastPHASE at low LD levels. Although fastPHASE performed similarly to MACH and IMPUTE at low LD levels, it showed lower ARs at medium and high LD levels, with AR differences of about 4% and 6%, respectively. The performance of PLINK and Beagle was inferior to MACH and IMPUTE under all LD levels. Further, Beagle was inferior to fastPHASE at low and medium LD levels, but slightly superior at a high LD level. PLINK was inferior to fastPHASE under all LD levels and inferior to Beagle at low and high LD levels, but slightly superior to Beagle at the medium LD level. Clearly, the LD level is a major determinant for imputation ARs for all methods.

**Figure 2:**
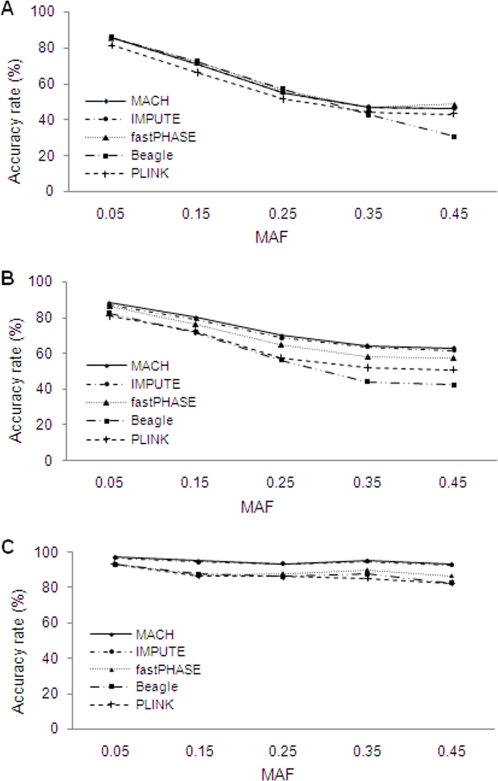
Effects of MAF of untyped SNPs on accuracy rates. The results are based on 90 reference haplotypes and the medium marker density (1 SNP per 6 kb). (a) Low LD level; (b) Medium LD level; (c) High LD level.

As shown in Figure 2, ARs decreased as MAF of untyped markers increased, particularly at low to intermediate LD levels. The influence of MAF on ARs was reduced at higher LD levels. For example, when the MAF interval increased from 0.05 to 0.45, ARs for MACH decreased from 85.4% to 46.4%, from 88.3% to 63.0%, and from 97.3% to 93.3%, under low, medium, and high LD levels, respectively. Similar trends were also seen for the other methods. When comparing the different methods, ARs achieved with MACH and IMPUTE were similar to one another at all LD and MAF conditions tested. ARs achieved with MACH and IMPUTE were generally superior to those achieved with fastPHASE, PLINK or Beagle, but the extent of these differences varied with different MAF and LD levels. Difference between MACH/IMPUTE vs. fastPHASE, PLINK and Beagle were greatest for medium LD levels.

**Figure 3:**
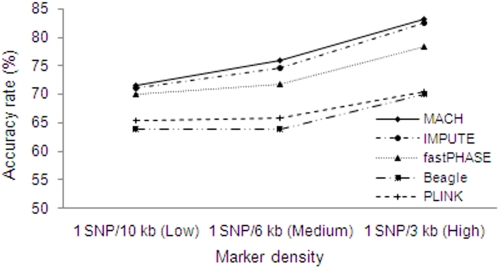
Effects of marker density on accuracy rates. The results are based on 90 reference haplotypes at the medium LD level. X-axis represents marker density: low marker density: one SNP per 10 kb; medium marker density: one SNP per 6 kb and high marker density: one SNP per 3 kb.

As expected, higher marker density led to better performances for imputation, as shown in Figure 3. For MACH, under a medium LD level with one SNP per 3 kb, the AR was 83.1%; when the density decreased to one SNP per 6 kb and one SNP per 10 kb, the ARs decreased to 75.9% and 72.4%, respectively. ARs attained with MACH and IMPUTE were again similar to one another, though ARs for IMPUTE were ∼1% below those of MACH. ARs attained with MACH were approximately 2–5%, 6–12% and 8–12% higher than those attained with fastPHASE, PLINK and Beagle, respectively.

**Figure 4:**
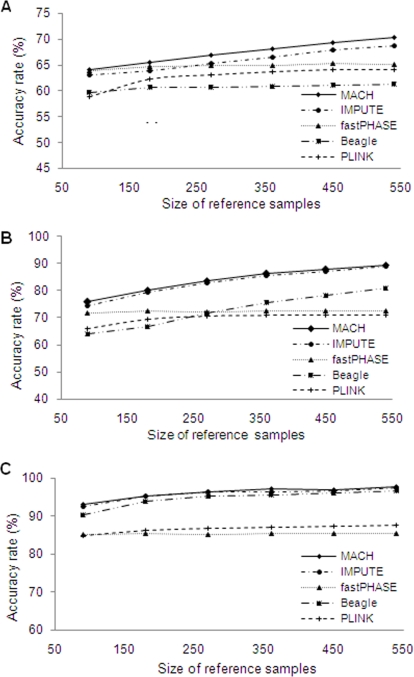
Effects of sample size of reference samples on accuracy rates under various conditions. (a) Low LD level and high marker density (one SNP per 3 kb); (b) Medium LD level and medium marker density (one SNP per 6 kb); (c) High LD level and low marker density (one SNP per 10 kb).

Figure 4 demonstrates the influence of the size of reference samples on ARs. For four of the five methods (MACH, IMPUTE, PLINK and Beagle), ARs increased as the size of reference haplotypes increased, while ARs for fastPHASE remained relatively constant. Again, MACH consistently had the highest AR, though the differences between MACH and some of the other methods were relatively minor under certain conditions. The difference between MACH and IMPUTE was greatest under the condition of low LD level and high marker density; differences between MACH and IMPUTE were barely discernible under other conditions. Beagle performed similarly to MACH and IMPUTE and better than fastPHASE and PLINK under a high LD level, but was markedly inferior to MACH and IMPUTE under medium and low LD levels. MACH and IMPUTE performed better than fastPHASE and PLINK under all conditions tested. The difference between MACH/IMPUTE and fastPHASE increased as the size of reference haplotypes increased because ARs for MACH and IMPUTE increased with increasing sample sizes, while ARs for fastPHASE remained nearly constant. fastPHASE performed better than Beagle under a low LD level and under a medium LD level when the size of reference haplotypes was below 270. With increasing size of reference haplotypes under a medium LD, however, ARs for Beagle increased while ARs for fastPHASE remained at approximately 72%. Consequently, when the reference sample size exceeded 270, Beagle's performance was superior to fastPHASE, with greater improvement as the reference sample size increased. PLINK performed better than Beagle under a low LD level and under a medium LD level when the size of reference haplotypes was below 270, but worse than Beagle under the other conditions. PLINK's performance was inferior to fastPHASE under low and medium LD level, but was superior to fastPHASE under high LD level.

### Analyses of Real Data

**Figure 5:**
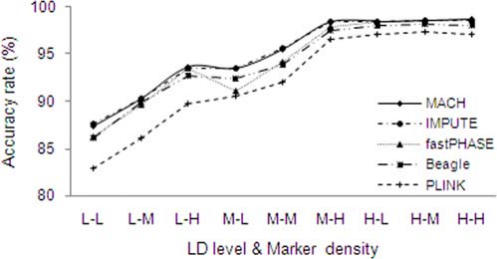
Performance of the imputation methods under various conditions using real data sets. Each label along x-axis represents a specific combination of LD level and marker density. Within each label, “L”, “M”, and “H” refer to, respectively, low, medium and high LD level when they are the first letter or marker density when they are the second letter.

**Figure 6:**
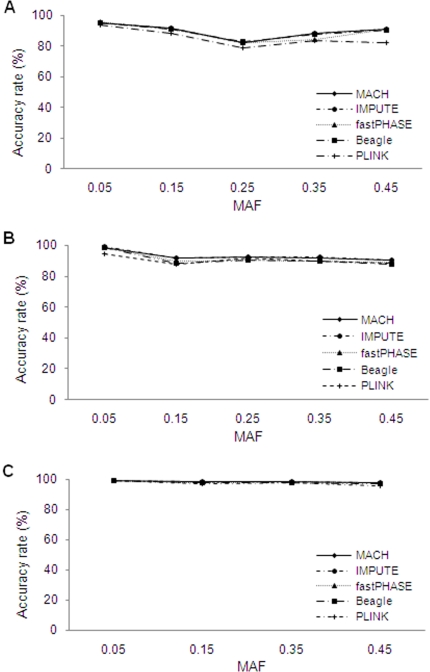
Effects of MAF of untyped SNPs on accuracy rates in real datasets. The results are based on the medium marker density (1 SNP per 6 kb). (a) Low LD level; (b) Medium LD level; (c) High LD level.

For the real data sets, LD and marker density influenced ARs in a similar manner to that attained with simulated data. For example, Figure 5 displays the rising trend of ARs with stronger LD levels and denser markers, and it is apparent that, in general, MACH and IMPUTE performed better than fastPHASE, Beagle and PLINK. Figure 6 shows the influence of MAF of untyped markers. Generally, MAF had little influence on accuracy in all the real regions. This pattern was similar to that for “high” LD regions in simulated data sets, illustrating that each of the considered real regions has an average LD level that is similar to or higher than those for the simulated regions with highest levels of LD; this was confirmed by calculating average *r* ^2^ and D′ across the regions. We also noticed an exceptional point under the low LD level (Figure 6A), where the AR under the 0.25 MAF interval was lower than that under MAF intervals of 0.35 or higher. We examined the data and found that the average values of *r* ^2^ and D′ under the 0.25 MAF were 0.40 and 0.86, while they were 0.47 and 0.94 under the 0.35 interval, and 0.46 and 0.94 under the 0.45 interval. This further analysis confirmed that the influence of MAF was ultimately largely caused by the patterns of LD.

Among the methods, MACH and IMPUTE yielded approximately equal accuracy rates, both of which performed better than fastPHASE, Beagle and PLINK.

### Running Time

All running times were obtained on a Linux cluster with 4 computation nodes, each having two Intel Xeon Quad-core processors and 7GB RAM. Times for imputing all missing genotypes for a sample of 100 individuals were recorded and converted to that on a single processor with a single core. All five methods completed a single imputation within 25 minutes. Running time was mostly influenced by sizes of the reference samples, with longer running times as reference sample sizes increased. Under a medium LD level and medium marker density as size of reference samples increased from 90 to 540, for example, the running time for MACH, IMPUTE, fastPHASE, PLINK and Beagle increased from 0.6 min to 15.5 min, from 0.50 min to 13.2 min, from 1.6 min to 6.5 min, from 0.15 min to 0.2 min and from 0.6 min to 0.7 min, respectively.

## Discussion

In this study, we investigated and compared the performance of five popular genotype imputation methods: MACH, IMPUTE, fastPHASE, PLINK and Beagle, under various conditions. Using both simulated and real data sets, we determined that factors such as LD level, MAF of untyped SNPs, marker density and size of reference haplotypes have varying effects on imputation accuracy rates. Specifically, stronger LD, lower MAF, or higher marker density lead to better ARs; greater size of haplotypes in the reference sample resulted in higher ARs for MACH, IMPUTE, PLINK and Beagle, but had little influence on ARs for fastPHASE. In comparing the different methods to one another, MACH and IMPUTE produced similar results that were generally better than fastPHASE, PLINK and Beagle. In addition, MACH performed better than IMPUTE under low LD levels and high marker densities.

One reason that missing genotypes can be imputed is that unrelated individuals from common ancestors usually share an extended haplotype over short regions [7], [12]. The approach by which haplotype sharing is captured differs for the five methods. In the following discussion, we did not summarize the model underlying PLINK since it was not accessible and not available at the time this study was performed. The remaining four methods all infer individual genotypes as mosaics from the set of background haplotypes by an HMM process [5], [7], [12], [27]. Despite their conceptual similarities, implemental distinctions between these methods have produced some differences in relative performance. fastPHASE relies on a fixed number of haplotype clusters to form underlying hidden states in the Markov Chain [12]. Provided that this number is correctly specified, fastPHASE should give an acceptably good performance. However the cluster number is usually restricted to a small value in real applications as a trade-off against computation cost, which makes this approach slightly inferior to the alternative approaches, under most conditions. Beagle uses a similar haplotype clustering approach to fastPHASE, but it allows the cluster number to dynamically change to better fit localized LD patterns exhibited by the data [7]. Nonetheless, empirical estimates of parameters in Beagle may bias specification of the model to some extent, particularly when the sequence exhibits a low average LD level. Both MACH and IMPUTE directly model genotypes on the set of haplotypes without clustering, and both of these methods appear to outperform fastPHASE and Beagle, which adopt haplotype clustering strategies [5], [27]. This improvement is probably attributable to their capacity to capture more information on haplotypic variation without clustering. IMPUTE explicitly specifies a set of reference haplotypes (e.g., haplotypes from the HapMap project), as the pool of hidden states of the Markov Chain, and infers haplotypes and missing genotypes in test samples according to these hidden states [5]. In contrast, MACH implicitly combines both reference and test samples together to estimate parameters and to update haplotypes for all individuals in turn by the Monte-Carlo procedure [27]. Generally, the two approaches have approximately equal performance. However, MACH performs a little better than IMPUTE under certain conditions as we show in the study, probably because it can make better use of the data by combining reference and test samples together to train model parameters.

Among various factors influencing imputation AR, the level of LD plays a central role for all methods. Stronger background LD patterns will improve imputation AR. The effects of marker density are essentially transformed into that of LD by the fact that denser markers usually cause stronger patterns of local LD. Thus, denser markers will also help improve imputation AR. The influence of MAF on imputation AR can be interpreted as ultimately caused by the level of LD. Our results demonstrated that a decrease in the MAF of untyped variants resulted in an increase in imputation AR. A lower MAF usually corresponds to a “younger” ancestral mutation, or a stronger LD with nearby markers, provided recombination plays a primary role in LD decay. To confirm this, we calculated average values of *r* ^2^ between typed and untyped markers under different MAF interval settings, for different LD regions in our simulation data. However, we did not find obvious relationship between *r* ^2^ and ARs. For example, in one of the simulated low LD region, average values of *r* ^2^ changed slightly around 0.006 regardless of MAF intervals. Nonetheless, when the level of LD was measured by D′, the trends in D′ change confirmed our explanations. For example, in the same region as above, average values of D′ decreased from 0.33 to 0.15 as MAF interval increased from 0.05 to 0.45. The discordance between *r* ^2^ and D′ was likely caused by the fact that calculation of *r* ^2^ was less sensitive to MAF than that of D′.

An interesting observation from our simulations is that MAF influences imputation AR in different patterns for regions with different LD levels. The influence of MAF was relatively minor for high LD regions, while it was considerably larger for low LD regions. One potential explanation for these findings is that in high LD regions the imputing AR is determined primarily by the high levels of LD between markers; the capacity for MAF to influence AR is greatly diminished under these circumstances. In low LD regions, on the other hand, markers with low MAF likely exhibit locally high levels of LD with nearby markers though the overall LD level across the entire region was low. The locally elevated LD level caused by the low MAF in low LD regions, results in much higher imputation AR than that attained with high MAF. In our simulations, D′ decreased from 0.71 to 0.41 as the MAF interval increased from 0.05 to 0.45 in high LD regions, whereas it decreased from 0.33 to 0.15 in low LD regions.

Larger samples will introduce extra information and will also produce more consistent estimates of measured parameters, resulting in generally improved AR for various methods. However, for fastPHASE, we observed that the number of reference haplotypes had little influence on imputation AR. One potential explanation for fastPHASE's insensitivity to reference sample sizes maybe its fixed small number of clusters. With low cluster numbers, increasing reference samples can only change parameter estimates within each cluster, but may not be able to capture the added haplotypic variation. Consequently, increasing reference samples has only a limited capacity to improve imputation AR. Increasing cluster number may resolve this issue, but that will be time-consuming and our simulations showed that the increase in the AR was not significant even when the cluster number increased from 20 to 100 (Data not shown). An alternative choice, that appears to improve AR, is to let the cluster number be determined dynamically by the data itself from the local context of sequence. This is the approach that Beagle adopted and, under these conditions, increasing the number of reference haplotypes improved imputation AR to a remarkable extent.

In the current study, test data and reference data were sampled from the same population, which is the basic assumption for most of the methods studied here. However, for many practical studies, these conditions do not apply; investigators often obtain their reference data from HapMap, which contains high-resolution haplotype information for a small number of relatively homogenous human populations. Importantly, several previous studies have demonstrated the feasibility of using homogeneous samples for reference data. For example, Marchini et al., imputed genotypes for a UK sample using CEU HapMap haplotypes, and the imputation AR was high [5]. Additionally, a worldwide survey of haplotype variation and LD patterns in 52 different populations demonstrated that there is considerable sharing of haplotype structure across groups and that locations of inferred recombination hotspots generally match across groups [28]. These studies support the conclusion that imputation can still be accurate even when there is mild heterogeneity between test samples and reference data.

In the current study, phases of the reference haplotype are assumed to be known, even though this is usually not true for real data. Inferring haplotypes from genotypes can introduce additional errors, with a consequent decrease in ARs for imputation using real data. Fortunately, it has been previously demonstrated that current haplotype inference programs (e.g. PHASE) can infer phasing information with high accuracy, thereby minimizing errors in subsequent imputation attributable to these inferred haplotypes [10], [11], [29].

One remaining issue related to imputed genotypes is how to apply imputed genotypes in subsequent analyses. In this study, the most likely genotypes were set as the imputed genotypes, but it is also possible to infer imputed genotypes from a posterior distribution provided by certain methods (such as the one based on HMM, IMPUTE) [5]. Both strategies, selecting the most likely genotype and selecting the posterior distribution of all possible genotypes, have demonstrated the capacity to improve power in follow-up association analysis [5], [6], [16]. However, comprehensive analyses appear to be warranted to better evaluate this issue.

## Materials and Methods

### Data Simulations

Haplotypes covering a 250 kb chromosomal region were simulated with uniformly distributed recombination rates across the region using the software Cosi [30] which is implemented under a coalescent model. From the pool of simulated haplotypes, a diploid individual was generated by combining two randomly selected haplotypes and a total of 100 individuals were sampled. SNPs with MAF less than 0.05 were excluded from further analyses. Two-hundred and fifty approximately equally spaced SNPs, corresponding to a density of one SNP per kb were selected as the base SNP set on which all subsequent analyses were based. Two types of samples were generated. The first type was a reference sample in which genotypes were known for all the 250 SNPs. A second sample was a test sample in which genotypes were known for only a proportion of these 250 SNPs. The number of SNPs with known (referred to as typed) genotypes in the test sample was determined by the marker density, and these SNPs were selected to be approximately equally spaced. The remaining SNPs in the based set were referred to as untyped SNPs and their genotypes would be inferred by imputation methods. The performance of a particular method was measured by imputation AR, which was defined as the number of correctly imputed genotypes divided by the total number of untyped genotypes.

Different parameter values were used to cover various biological conditions. Three recombination rates (between neighboring sites per generation): 1.0e-7, 1.0e-8 and 1.0e-9 were used to represent low, medium and high levels of LD, respectively, consistent with previous studies [31]. To make the definition of LD levels clearer, we calculated the average *r* ^2^ and D′ values between adjacent SNPs across the whole 250 kb sequence, which were 0.03 and 0.46, 0.15 and 0.83, 0.31 and 0.98 for regions with low, medium and high levels of LD, respectively. To select typed SNPs, three marker densities: one SNP per 3 kb, per 6 kb, and per 10 kb, were assumed, corresponding to approximate 83, 41 and 25 typed SNPs, respectively, in the study region. To mimic the practical situation where external information is available, such as known phased haplotype data sets from the HapMap project, we generated reference samples with different sample sizes (90, 180, 270, 360, 450 and 540) with all the 250 SNPs typed in simulations. In addition, effects of MAF were studied by binning untyped SNPs into one of five equal-width intervals between 0.0 and 0.5 (0.05, 0.15, 0.25, 0.35 and 0.45). For each parameter setting, 1000 replications were performed and the average AR was reported.

### Real Data Sets

Phased haplotype data for individuals in the HapMap CEU sample were downloaded (HapMap rel#21) from the website http://www.hapmap.org/downloads/phasing/2006-07_phaseII/phased/. Monomorphic SNPs were deleted. Although there were 30 trios genotyped, we only selected 60 unrelated parents to form a sample of 120 haplotypes. Based on estimated recombination rates given on the HapMap website, three 250 kb chromosomal regions on chromosome 22 (35109556∼35341653, 22246455∼22505676, 30809496∼31058109), with average recombination rates of 6.55 cM/Mb, 1.09 cM/Mb, and 0.24 cM/Mb, were selected, corresponding to regions with low, medium and high levels of LD, respectively. These regions contained 250, 242 and 250 SNPs, respectively. We also calculated the average *r* ^2^ and D′ values between adjacent SNPs across the whole 250 kb sequence, which were 0.42 and 0.93, 0.38 and 0.97, 0.69 and 0.98 for regions with low, medium and high levels of LD, respectively. To obtain the reference samples, we adopted a cross-validation procedure, in which 100 of 120 haplotypes were randomly selected, and the remaining 20 haplotypes were assigned into the test samples. In addition, for the test samples, we used the same scheme as for the simulated data and selected typed SNPs based on three marker densities (one SNP per 3 kb, per 6 kb and per 10 kb). 1000 iterations were then performed for each setting and average ARs were reported.

### Genotype Imputation Methods

Five popular imputation methods were investigated in this study: MACH, IMPUTE, fastPHASE, PLINK and Beagle. These methods are briefly described below.

#### MACH

MACH v 1.0.10 implements a Markov Chain based algorithm [13], [27] to infer possible pairs of haplotypes for each individual's genotypes (including untyped genotypes). It defines a series of indicators (S) to denote unobserved states underlying unphased genotypes and models S as a Markov Chain. The algorithm begins by randomly assigning a pair of haplotypes to each individual that is consistent with the observed genotypes. For untyped sites, alleles are assigned according to their population frequencies. Then it updates haplotype configurations by using the current set of haplotype estimates for all individuals as templates, and sampling S using the Markov Chain. It repeats the update procedure a number of times and counts how often a genotype is sampled at a particular position. In this study we used the command *mach –d sample.dat –p sample.ped –h sample.hap –s sample.snps -–rounds 50 -–greedy -–geno -–profix filename* to impute untyped genotypes. Under this standard setting, MACH can work with very high accuracy at the cost of intensive computation. Alternatively, it can run faster without much loss of accuracy by using a two-stage process (using a single set of estimates for the crossover and error rate map and, conditional on these, to find the most likely genotypes). However, as both simulated and real data sets had moderate sizes in our study, we ran MACH with the standard option to get the highest AR. The most likely genotype is the one that is sampled most frequently. In this study, the number of iterations of the Markov Chain was set to 50 to assure a reliable result. MACH is available at http://www.sph.umich.edu/csg/abecasis/MACH/download/.

#### IMPUTE

IMPUTE v 0.2.1 is a hidden Markov Model based algorithm [5]. It treats the sequence of pairs of known haplotypes as hidden states and models the sequence of hidden state change along the sequence with switching rates depending upon a recombination map estimated from the reference data. Then based on known haplotypes, it predicts untyped genotypes. IMPUTE was run with default command *impute –h sample.hap –l sample.legend –m sample.map –g sample.geno –Ne 11000 –o output –i filename* to impute untyped genotypes. As IMPUTE outputs posterior probability of each potential genotype, to facilitate the comparison, the imputed genotype was defined as the one that had the highest posterior probability. This program is available at http://www.stats.ox.ac.uk/marchini/software/gwas/impute.html.

#### fastPHASE

fastPHASE v 1.2.3 is a haplotype clustering algorithm [12]. It assumes that haplotypes in a population cluster into groups over a short region and allows cluster memberships to change continuously along the chromosome based on a HMM. First, missing genotypes are sampled based on allele frequencies estimated from reference haplotypes, and then an Expectation-Maximization (EM) algorithm is used to estimate parameter values. Based on estimated parameters, missing genotypes are inferred. In this study, the number of clusters was set to 20, and haplotype estimation was turned off by using option -H-4 to save time. The command we used was *fastPHASE –K20 –T20 –C25 –H-4 –Z –ooutput –n –brefname geno*. Also fastPHASE can determine the number of clusters via cross-validation procedure, but this added considerably to the running time. fastPHASE is available at http://depts.washington.edu/ventures/UW_Technology/Express_Licenses/-fastPHASE.php.

#### PLINK

PLINK v 1.03 is essentially based around the concept of multi-marker tagging [32]. The detailed description of the algorithm implemented in PLINK was not available when we prepared this manuscript. We just used the default parameter setting to output the posterior probabilities of each genotype and the command was: *plink -–bfile filename -–all -–proxy-impute all -–proxy-verbose -–make-bed -–out outname*. We defined the imputed genotype as the one that had the highest posterior probability. PLINK is available at http://pngu.mgh.harvard.edu/purcell/plink/download.shtml.

#### Beagle

Beagle v 2.1.3 is a haplotype clustering based algorithm [7]. First it uses the localized haplotype cluster model to cluster haplotypes at each marker and then defines an HMM to find the most likely haplotype pairs based on the individual's known genotypes. Then the most likely genotype at untyped loci can be generated from final haplotype pairs. Both Beagle and fastPHASE use an HMM approach to cluster haplotypes, but they have some slight differences. First, fastPHASE uses an EM algorithm to estimate parameters for cluster configurations, while Beagle uses empirical frequencies as parameters. Second, fastPHASE fixed the number of clusters in the model while Beagle can vary the number of clusters at each locus to model the data. As recommended by the authors, nsamples (s) (the number of haplotype pairs to sample for each individual) was set to a value so that the product of “nsamples” and the number of individuals is between 2000 and 4000. The command we used was *Java –jar beagle.jar unphased = geno missing = x nsample = s out = output*. This algorithm is available at http://www.stat.auckland.ac.nz/browning/beagle/beagle.html.
